# Impact of socioeconomic status on the 90- and 365-day rate of revision and mortality after primary total hip arthroplasty: a cohort study based on 103,901 patients with osteoarthritis from national databases in Denmark

**DOI:** 10.1080/17453674.2021.1935487

**Published:** 2021-06-04

**Authors:** Nina M Edwards, Claus Varnum, Søren Overgaard, Alma B Pedersen

**Affiliations:** a Department of Clinical Epidemiology, Aarhus University Hospital; b Department of Orthopaedic Surgery, Lillebaelt Hospital—Vejle, and Department of Regional Health Research, University of Southern Denmark; c Danish Hip Arthroplasty Register; d Department of Orthopaedic Surgery and Traumatology, Copenhagen University Hospital, Bispebjerg, University of Copenhagen; and Department of Clinical Medicine, Faculty of Health and Medical Sciences, Denmark

## Abstract

**Background and purpose** — Socioeconomic inequality in health is recognized as an important public health issue. We examined whether socioeconomic status (SES) is associated with revision and mortality rates after total hip arthroplasty (THA) within 90 and 365 days.

**Patients and methods** — We obtained SES markers (cohabitation, education, income, and liquid assets) on 103,901 THA patients from Danish health registers (year 1995–2017). The outcomes were any revision (all revisions), specified revision (due to infection, fracture, or dislocation), and mortality. We used Cox regression analysis to estimate adjusted hazard ratio (aHR) of each outcome with 95% confidence interval (CI) for each SES marker.

**Results** — Within 90 days, the aHR for any revision was 1.3 (95% CI 1.1–1.4) for patients living alone vs. cohabiting. The aHR was 2.0 (CI 1.4–2.6) for low-income vs. high-income among patients < 65 years. The aHR was 1.2 (CI 0.9–1.7) for low liquid assets among patients > 65 years. Results were consistent for any revision within 365 days as well as for revisions due to infection, fracture, and dislocation. The aHR for mortality was 1.4 (CI 1.2–1.6) within 90 days and 1.3 (CI 1.2–1.5) within 365 days for patients living alone vs. cohabiting. Low education, low income, and low liquid assets were associated with increased mortality rate within both 90 and 365 days.

**Interpretation** — Our results suggest that living alone, low income, and low liquid assets were associated with increased revision and mortality up to 365 days after THA surgery. Optimizing medical conditions prior to surgery and implementing different post-THA support strategies with a focus on vulnerable patients may reduce complications associated with inequality.

Socioeconomic inequality in health is increasingly recognized as an important public health issue (Agabiti et al. 2007). Socioeconomic status (SES) is associated with access to total hip arthroplasty (THA) and with greater vulnerability to complications after THA, all in favor of high status (Agabiti et al. 2007, Weiss et al. 2019, Edwards et al. 2021). However, few studies have investigated the impact of SES on the risk of revision and mortality and they all present contradicting results, from showing that low SES was associated with a higher risk of early mortality after a THA, to finding no association among SES, revision, and mortality (Mahomed et al. 2003, Agabiti et al. 2007, Jenkins et al. 2009, Peltola and Järvelin 2014, Maradit Kremers et al. 2015). Previous research is limited by assessing SES by only a single marker, by lack of adjustment for important confounders and hospital factors, and by a lack of clinically relevant differentiation between time periods regarding risk assessment. Disparities in the risk of revision and mortality are important as social inequality is a growing problem in Denmark despite universal tax-supported healthcare (Sundhedsstyrelsen and Folkesundhed 2020). Even though the inequality in Denmark and most Nordic countries is less than the inequality seen in the United States (OECD 2019), our hypothesis is that even within this smaller spectrum of inequality, we shall find SES disparities concerning the risk of revision and mortality. By examining and identifying these disparities, we may be able to improve patient outcome by more focused risk assessment with proper counselling and optimization of medical risk factors prior to surgery and by implementing different postoperative strategies.

We examined the association between multiple SES markers and the rates of any revision as well as revisions due to infection, fracture, and dislocation, and mortality within 90 and 365 days after THA.

## Patients and methods

### Study design and setting

All Danish citizens are assigned a unique civil registration number at birth, which is included in all Danish registries, allowing for unambiguous record linkage on an individual level between multiple registers and almost complete long-term follow-up of all Danish inhabitants (Schmidt et al. 2014).

For this study, we linked data from the Danish Civil Registration System (DCRS), which tracks vital status, migrations, and cohabitation status (Schmidt et al. 2014); the Danish Hip Arthroplasty Registry (DHR), which holds information on primary THA and revision surgeries with high completeness (91–98%) (Gundtoft et al. 2016); the Danish National Patient Registry (DNPR), which contains discharge dates and diagnoses from all hospitalizations since 1977, and outpatient clinic and emergency room contacts since 1995 (Schmidt et al. 2015); and Statistics Denmark, which contains detailed individual-based information on socioeconomic characteristics for all Danish citizens.

This study is reported following the STROBE and RECORD guidelines.

### Study population and outcome

We conducted a population-based cohort study using prospectively collected data from the DHR. We identified all patients over the age of 45 undergoing primary THA in Denmark from January 1, 1995 to December 31, 2017 with the primary diagnosis idiopathic osteoarthritis (OA). Only the first THA during 1995–2017 was included in the study cohort; if the patient received bilateral THA on the same date, only the right THA was included in the study.

The outcomes were revision divided into any revision or revision specified as due to infection, fracture, or dislocation. Revisions were identified in the DHR and defined as any later surgical procedure involving the primary THA, including change of any component or debridement without removal of any part of the prosthesis (Gundtoft et al. 2016). In addition, we studied mortality, defined by date of death due to any cause from the Danish Civil Registration System. All outcomes were evaluated within 90 or 365 days after the date of the primary THA procedure.

### Socioeconomic status

For each THA patient, we retrieved information on SES using the following markers: cohabitating status, highest obtained education, mean family income, and mean family liquid assets.

Cohabitating status was classified into 2 categories: living alone and cohabiting. Highest obtained education was classified into 3 categories: low, defined as none or elementary school; medium, defined as more than elementary school, but less than university completed; and high, defined as university degree completed. Since a large proportion of the THA patients are senior citizens (> 65 years of age) with a state pension, family liquid asset was used as an SES marker in patients > 65 years of age, whereas family income was used as an SES marker in patients < 65 years of age (Robert and House 1996). This provides a more accurate estimate of overall socioeconomic stratification than using income and liquid assets through all ages (Robert and House 1996). To account for yearly variations in income and liquid assets, we calculated the average yearly total income and liquid assets in the 5 years prior to primary THA surgery for the patient and the patient’s cohabiting partner. According to tertiles, the family mean income and liquid assets were categorized into 3 groups of increasing amount: low, medium, and high (income: < €31,400, €31,400–49,400, ≥ €49,400; liquid assets: < €82,653, €82,653–240,068, ≥ €240,068, respectively).

### Covariates

We collected information on the following variables recorded at the time of primary THA: 1) From the DCRS, we collected information on age and sex. 2) Data on comorbidities was obtained from the DNPR. Based on discharge diagnoses codes 10 years before primary THA, we calculated the Charlson Comorbidity Index (CCI) score adapted to administrative data for every patient. We defined 3 levels of comorbidity: a CCI score of 0 (low) was given to patients with no known comorbidities included in the CCI; a CCI score of 1–2 (medium); and a CCI score of 3 or more (high) (Charlson et al. 1987, Schmolders et al. 2015).

### Statistics

We tabulated the patients’ characteristics by SES markers and calculated the cumulative incidence with 95% confidence intervals (CI) of revisions, starting follow-up at the date of primary THA and treating death as competing risk. Cumulative incidence curves were plotted for any revision; and mortality was calculated within 1 year by cohabitation, education, income, and liquid assets.

We used a Cox proportional hazard model to calculate time to event estimating hazard ratios (HRs) with CI for each SES marker and evaluated with a distinction within 90 days and 365 days after THA. The association between the SES markers and revision rate was assessed by using a multilevel model with inclusion of random effects into the Cox proportional hazards model. Subjects who are nested within the same higher-level unit are likely to have outcomes that are correlated with one another. This within-cluster homogeneity may be induced by unmeasured cluster characteristics, here hospitals, that affect the outcome. The Cox model is enhanced when random effects are incorporated through terms to account for within-cluster homogeneity in the outcomes and allows the intercept to vary randomly across clusters. This denotes an increased or decreased hazard by clustering at hospital level (Austin 2017).

The HRs were adjusted for potential confounders: age, sex, calendar year, and CCI. We considered the SES markers’ interdependency, since a mutual adjustment for each SES marker would assume no effect of the common aspects of the SES markers, but that all effects are due to the unique characteristics of the different SES markers (Green and Popham 2019). By applying the directed acyclic graph method, only cohabiting status was evaluated as a true confounder when calculating the HR for revision by income and liquid assets (Figure 1, see Supplementary data). Forest plots were plotted including HRs for revision and mortality for each SES marker. The assumption of proportionality of hazards was fulfilled by calculation of scaled Schoenfeld residuals and by graphically assessing by plotting the residuals against time.

The study period was from 1995 to 2017. We performed a sensitivity analysis to account for yearly variations. The period was divided into 2: 1995–2005 and 2006–2017.

The statistical analyses were performed in STATA version 15 (StataCorp, College Station, TX, USA) and R version 3.6.1 (R Foundation for Statistical Computing, Vienna, Austria) with use of the coxme package (https://cran.r-project.org/web/packages/coxme/index.html) to compute the Cox model and to estimate the HRs.

### Ethics, funding, and potential conflicts of interest

The study was approved by the Danish Data Protection Agency (journal number 2015-57-0002) and Aarhus University (journal number 2016-051-000001). We would like to acknowledge the support from Helsefonden, the Orthopaedic Research Fund, the AP Møller Fund, and the Aase and Ejnar Danielsens Fund. The funders had no role in the study design, data collection and analysis, or in the preparation of the manuscript. The authors report no conflict of interest.

## Results

### Study population

**Figure 2.**
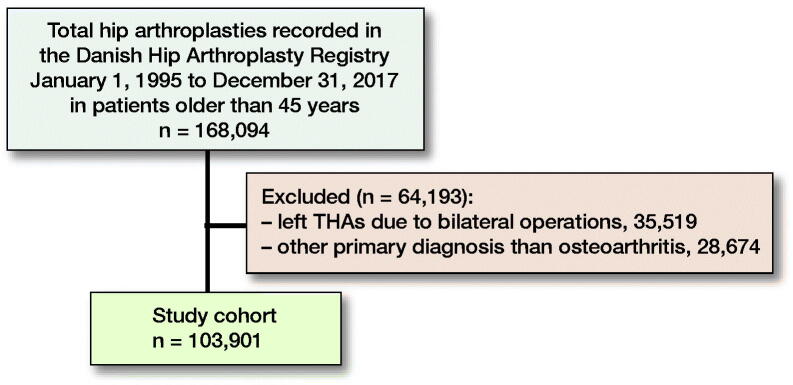
Flowchart of total hip arthroplasty (THA) cohort.

**Figure 3.**
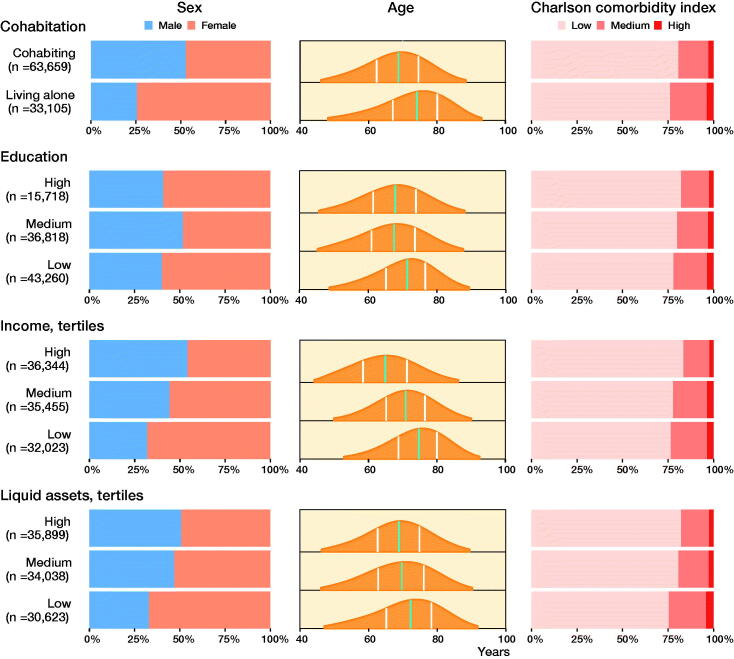
Demographics with distribution of sex, age and Charlson comorbidity index score in the 4 SES markers. Sex and Charlson comorbity index score distribution is given in percent on the x-axis. Age distribution is shown with age on the x-axis, green line is median age and white lines marks the first and third quartile.

We identified 168,094 THAs in the DHR. We excluded 35,519 left THAs due to bilateral THA surgery (keeping the right THA), and 28,674 THA patients due to a primary diagnosis other than hip OA. The final study population included 103,901 THA patients (Figure 2). The median follow-up was 7 years (0–23) for revision and 9 years (0–23) for mortality. Some patient characteristics were unevenly distributed across the different SES groups. The distribution of females was between 46% and 74%, with the highest proportion when patients lived alone, had the lowest and highest education, had the lowest income, and had the lowest liquid assets. The mean age distribution in the different SES groups was 65–74 years of age, lowest in the high-income category and highest in the lowest income category (Figure 3 and Table 1, see Supplementary data).

### Socioeconomic status and revision

**Figure 4.**
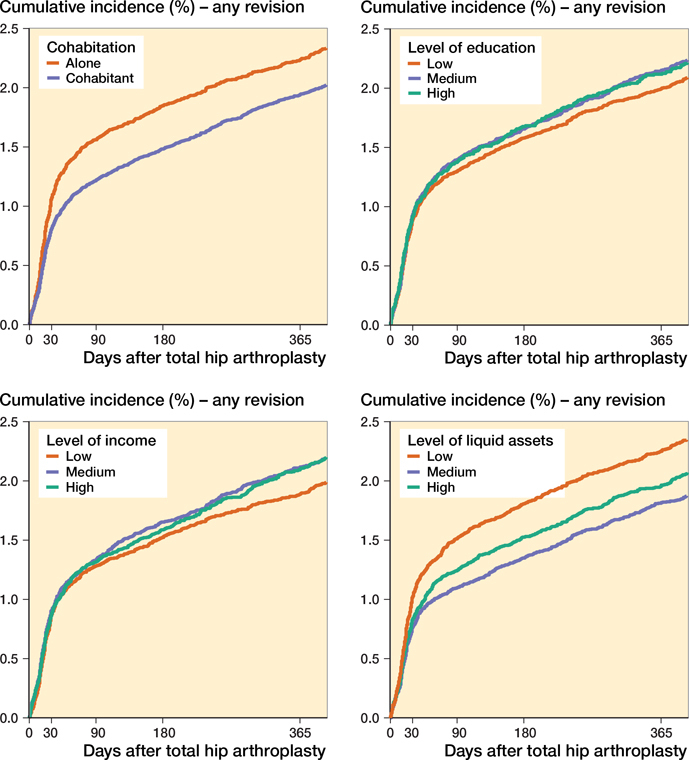
Cumulative incidence of any revision for the 4 SES markers.

Within 90 days after primary THA, 1,364 (1.3% of the study population) revisions were identified, whereas within 365 days, 2,092 (2.0% of the study population) revisions were identified. The cumulative incidence of any revision at 1 year was highest among patients who lived alone (2.2%; CI 2.1–2.4), had the highest education (2.1%; CI 1.9–2.9), had the highest income (2.1%; CI 2.0–2.3), and had the lowest liquid assets (2.3%; CI 2.1–2.4) (Figure 4 and Table 2, see Supplementary data). In the stratified analysis, the cumulative incidence from 1995 to 2005 varied little in the SES categories. In the stratification from 2006 to 2017, the trends from the main analysis were enhanced except within level of income. Here the cumulative incidence of any revision was highest among patients who had the lowest income (Figures 5 and 6, see Supplementary data).

**Figure 7.**
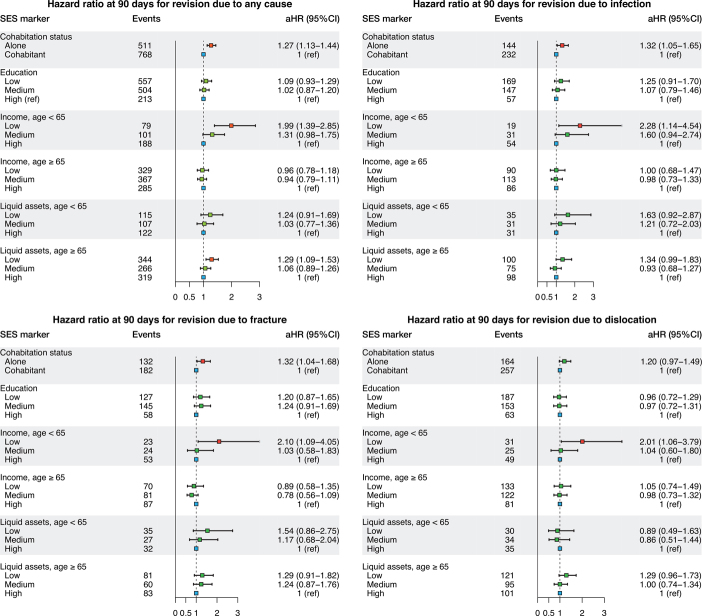
Hazard ratios for revision due to any cause, infection, fracture, and dislocation at 90 days. Hazard ratios are adjusted for age, sex, calendar year, and CCI. Income and liquid assets were also adjusted for cohabiting status.

The 90-day results showed that living alone was associated with higher rates than cohabiting where the adjusted HRs were 1.3 (CI 1.1–1.4) for any revision, 1.3 (CI 1.1–1.7) for revision due to infection, 1.3 (CI 1.0–1.7) for revision due to fracture, and 1.2 (CI 1.0–1.5) for revision due to dislocation. Education was not associated with 90-days’ revision rate. In contrast, the HR was 2.0 (CI 1.4–2.9) for any revision for patients with low income compared with patients with high income in the age groups under 65 years. This association was not present in the age group over 65 years. Low liquid assets were associated with higher rates of any revision than high liquid assets. This was seen both among patients younger than 65 years of age (HR 1.2; CI 0.9–1.7) and those older than 65 years of age (HRs 1.3; CI 1.1–1.5) (Figure 7 and Tables 3 and 4, see Supplementary data). In the stratified analysis, the HRs from 1995–2005 and 2006–2017 periods were similar to the HRs from the overall 1995–2017 period (Tables 5 and 6, see Supplementary data).

Similar results were seen for the 365-day adjusted HR for any revision and for revisions due to infection, fracture, and dislocation (Figure 8 and Tables 3 and 4, see Supplementary data).

### Socioeconomic status and mortality

**Figure 9.**
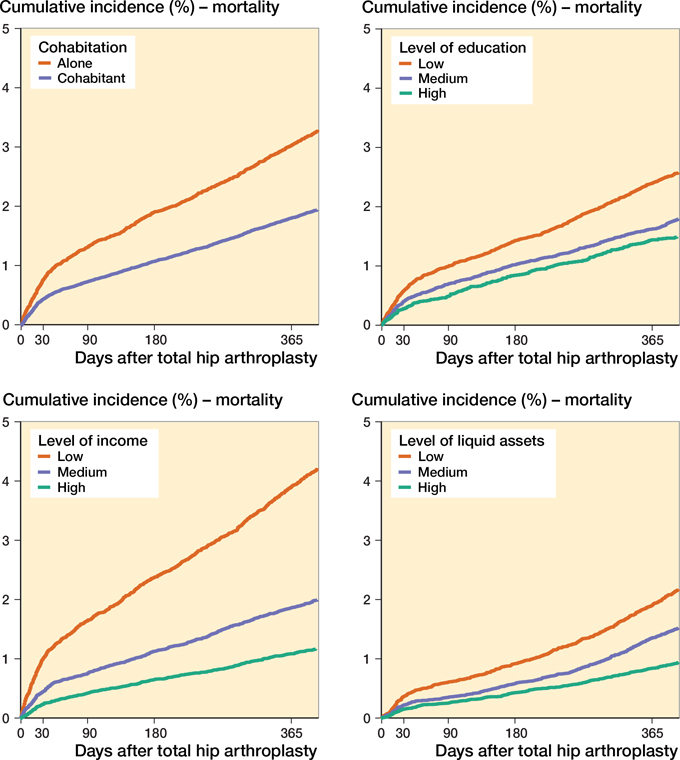
Cumulative incidence of mortality for the 4 SES markers.

Within the first 90 days after surgery, 942 died (0.9% of the study population), whereas within the first 365 days, 2,251 died (2.2% of the study population). The cumulative incidence for mortality at 365 days was highest when patients lived alone (3.0%; CI 2.8–3.2), had the lowest education (2.4%; CI 2.3–2.5), had the lowest income (3.9%; CI 3.7–4.1), and had the lowest liquid assets (1.9%; CI 1.8–2.1) (Figure 9 and Table 2, see Supplementary data).

**Figure 10.**
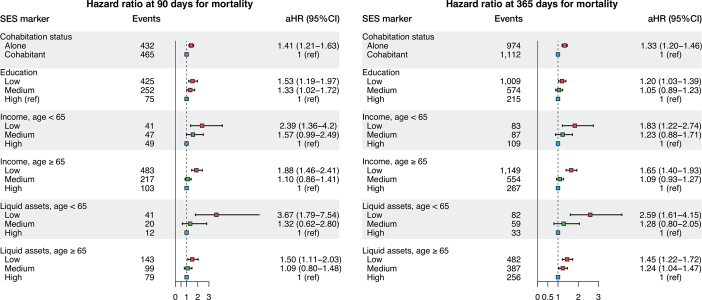
Hazard ratios of mortality for the 4 SES markers. Hazard ratios are adjusted for age, sex, calendar year, and CCI. Income and liquid assets were also adjusted for cohabiting status.

The adjusted HR for the 90-day mortality was 1.4 (CI 1.1–1.6) among patients living alone compared with cohabiting patients. The adjusted HR for the 90-day mortality was 1.5 (CI 1.2–2.0) for patients with low education compared with patients with high education. Among patients younger than 65 years of age, the adjusted HR for the 90-day mortality was 2.4 (CI 1.4–4.2) for patients with low income compared with patients with high income. Similar results were seen in the age group over 65 years. Low liquid assets were associated with higher 90-day mortality rates than high liquid assets. This was seen both among patients younger than 65 years of age (HR 3.8; CI 1.8–7.5) and those older than 65 years of age (HRs 1.5; CI 1.1–2.0). Similar results were obtained for mortality within 1 year (Figure 10 and Table 7, see Supplementary data).

## Discussion

In this large nationwide cohort study of 103,901 patients, we observed substantial socioeconomic inequality in terms of revision and death after THA. Living alone, low income, and low liquid assets were associated with increased rate of revision and mortality after both 90 days and 365 days. In addition, low education was associated with increased mortality rate.

### SES markers

Social support can be defined as those resources in a person’s environment that enable the person to deal with life’s physical and psychological stress; a crude measurement may be obtained by dichotomizing patients according to cohabitation status (Brembo et al. 2017). We found a higher rate of revision due to fracture, infection, and dislocation when patients were living alone. This was seen especially within the first 90 days, where social support is important to maintain the household and everyday living arrangements and thereby reduce risk of falling. In line with this, the presence of social support is, according to Brembo et al. (2017), associated with improved bodily pain and physical function outcomes in general, and a study by Weiss et al. (2019) found increased risk of readmission when living alone. Our findings indicate that low education, which is a non-modifiable factor, was only weakly associated with increased revision rate. This contrasts with findings by Maradit Kremers et al. (2015) and Weiss et al. (2019). Others argue that there is a positive correlation between education and psychological health and well-being as well as income and standard of living, a correlation that nurtures the expectation of a correlation between education and risk of revision (Edgerton et al. 2012, Weiss et al. 2019). Education is a widely used international marker of socioeconomic status, since it captures the long-term influence of both early life circumstances on adult health and the influence of adult resources on health. Moreover, it remains relatively constant throughout life (Galobardes et al. 2006). However, recent decades have seen considerable changes in educational opportunities for specific subgroups (Galobardes et al. 2006). This applies in particular to women and the elderly, which leads to an over-representation of these sub-groups among the less educated. These same sub-groups have a decreased risk of revision (Bayliss et al. 2017), which would lead to an underestimation of our results and hence explain the weak association seen in our study.

An interaction between health behavior and the different SES markers is evident through a variety of mediating factors such as lifestyle factors (Robert and House 1996). With higher income and liquid assets come the possibility of better diet, better rehabilitation, and better suited accommodation. Our findings of an association between low income in the age group under 65 and low liquid assets in the age group over 65 and a higher rate of revision contrast with previous findings (Peltola and Järvelin 2014). They describe the opposite effect of income. However, they found a U-formed association with a higher HR in the lower income categories as well in the higher income categories. This supports our findings, and the missing stratification in age in their study may explain the U-formed tendency. Most studies have a median age of +65 when examining effects in respect of income (Agabiti et al. 2007, Peltola and Järvelin 2014, Maradit Kremers et al. 2015, Weiss et al. 2019); however, an age of +65 is above the age of retirement, leaving a population with an income with less fluctuation and perhaps skewed values. Dividing the results concerning income and liquid assets into 2 age groups allows us to assess the effect as regards the true subpopulation, as we did in the present study.

### Mortality

Our findings of an association between the low strata in all of our SES markers and a higher mortality in both the 90-day and the 365-day follow-up are in accordance with findings in earlier studies and are similar to the associations reported for the general population (Maradit Kremers et al. 2015, Ullits et al. 2015, Weiss et al. 2019). In particular, the findings by Weiss et al. (2019) support this, since they have a similar study setup and data quality. However, they have a different aim and chose to mutually adjust for the SES markers. Nearly half of the deaths that occurred within the first year occurred within the first 90 days after surgery. Some of this 90-day mortality would be caused by the surgery, as terminally ill patients are rarely offered elective surgery. This inequality in mortality even in this short period of time after THA indicates underlying diseases, health-care behavior, or social network as possible explanations rather than surgery itself.

### Methodological considerations

The strengths of our study include prospective data collection where information on SES markers was collected from registers on an individual level with few missing data (data not shown as counts < 5 for each marker). Including liquid assets as an SES marker is also a clear advantage compared with other studies, because it provides us with a more accurate estimation of SES for individuals over the age of 65. We included a novel approach to evaluate the relationship between the rate of revision and SES by assessing the rate using a multilevel model with inclusion of random effects in the Cox proportional hazards model. Conventional survival models do not account for the loss of independence that arises from the clustering of patients in higher-level units (Austin 2017). Another strength of our study is the calendar year stratification. From this we can conclude that the HRs are not driven by changes in SES markers over time, but remain despite differences seen in the cumulative incidence.

A limitation of our study is the contradictory results regarding cumulative incidence and HR. Because of the differences in the risk of THA-related mortality between the SES groups, different numbers of patients are at risk of revision over time in the different groups. These risks are implicitly incorporated when modelling the cumulative incidence function, whereas the Cox model considers the risk of revision only among those still at risk, giving opposing results (Logan et al. 2006). This paradox also hampers interpretation of the results, as the hazard cannot be directly translated into relative risk. Another limitation is that we have no information regarding lifestyle-related confounders, such as BMI, smoking, alcohol, and physical activity. These confounders differ between socioeconomic strata, influencing the observed associations between socioeconomic variables and our outcome (Weiss et al. 2019).

Risk factors for revision and mortality are heart failure, diabetes, obesity, anemia, and malnutrition, and, as seen in Figure 2, the comorbidity burden in the most disadvantaged patients is higher. Some of the effects seen may therefore be driven by pre-existing risk factors, and proper optimization of these medical conditions prior to surgery may therefore minimize the inequality seen (Baek 2014, Romero et al. 2017). The mechanisms explaining the effect of SES on health outcomes are complex and not always clear. However, we examined important socioeconomic factors and found that the correlation between our markers and the rate of revision and mortality was not consistent, showing that treating these markers as indicators of the same fundamental cause ignores their sometimes sizeable independent and distinct contributions to health (Geyer et al. 2006).

In conclusion, we found socioeconomic inequality in the rates of revision and mortality after THA. Living alone, low income, and low liquid assets were associated with increased revision and mortality up to 365 days after THA surgery and were associated with inequality both when examining the 90-day and the 365-day hazard rate. Living alone is the most noticeable marker, as this is an easily measured factor with multiple options for intervention. We cannot change patients’ cohabitation status, but by optimizing pre-existing risk factors prior to surgery in patients living alone, and offering better rehabilitation to these patients, we may secure a better minimal level of function, improving their outcome and reducing complications associated with inequality in this respect. Another aspect is when assessing patient frailty and evaluating implant choice prior to surgery, the surgeon should also consider patient SES. However, our study does not support choosing any particular implant over another. Current evidence hence supports implementation of different pre- and post-THA strategies for patients who are living alone and in a lower SES group in general and for patients with increased vulnerability in particular. Further research is needed to clarify the mechanism leading to increased revision and mortality rates among patients with lower SES status.

## Supplementary data

Tables 1–7 and Figures 1, 5, 6, and 8 are available as supplementary data in the online version of this article, http://dx.doi.org/10.1080/17453674.2021.1935487
